# AN MDS 3.0 BEHAVIORAL INDICATOR: RELIABILITY AND VALIDITY WHEN MEASURING BEHAVIOR SYMPTOMS OF DEMENTIA IN CLCS

**DOI:** 10.1093/geroni/igz038.2352

**Published:** 2019-11-08

**Authors:** Kim Curyto, Jenefer M Jedele, Jennifer J Mach, David C Mohr, Laura O Wray, April Eaker, Orna Intrator

**Affiliations:** 7 GECDAC, Canandaigua VA Medical Center & Department of Public Health Sciences, University of Rochester, Rochester, New York, United States; 1 Center for Integrated Healthcare, VA Western New York Healthcare System, Batavia, New York, United States; 2 Serious Mental Illness Treatment Resource and Evaluation Center, Office of Mental Health and Suicide Prevention, Department of Veterans Affairs, Ann Arbor, Michigan, United States; 3 Serious mental Illness Resource and Evaluation Center, Office of Mental Health and Suicide Prevention, Department of Veteran Affairs, Ann Arbor, Michigan, United States; 4 VA HSR&D Center for Healthcare Organization and Implementation Research, Boston VA Healthcare System, Boston, Massachusetts, United States; 5 VA Center for Integrated Healthcare, VA Western NY Healthcare System (116N), Buffalo, New York, United States; 6 Center for Integrated Healthcare, VA Western NY Healthcare System, Buffalo, New York, United States

## Abstract

Persons with dementia frequently demonstrate behavior symptoms of dementia (BSD), associated with poorer outcomes. A measure of BSD was created for routine use in VA Community Living Centers (CLCs). Reliability and validity of Minimum Data Set (MDS 3.0) behavior items was established using exploratory factor analysis and a multitrait, multimethod correlation matrix. 385 CLC residents with BSD were assessed using validated measures of BSD, depression, and anxiety, and team ratings of the frequency and severity of target behaviors identified for intervention. Factor analysis on MDS items closest to baseline resulted in two stable factors. MDS behavior factors related to validated clinical measures in predicted ways at baseline and post-intervention. MDS distress behavior factor sensitivity to change was evaluated by using change score correlations with validated clinical measures. The MDS distress behavior factor can be used routinely, evaluate the impact of intervention effectiveness, and provide quality improvement feedback.

